# Signaling Inhibitors Accelerate the Conversion of mouse iPS Cells into Cancer Stem Cells in the Tumor Microenvironment

**DOI:** 10.1038/s41598-020-66471-2

**Published:** 2020-06-22

**Authors:** Juan Du, Yanning Xu, Saki Sasada, Aung Ko Ko Oo, Ghmkin Hassan, Hafizah Mahmud, Apriliana Cahya Khayrani, Md Jahangir Alam, Kazuki Kumon, Ryo Uesaki, Said M. Afify, Hager M. Mansour, Neha Nair, Maram H. Zahra, Akimasa Seno, Nobuhiro Okada, Ling Chen, Ting Yan, Masaharu Seno

**Affiliations:** 1 Department of Medical Bioengineering, Graduate School of Natural Science and Technology, Okayama University, Okayama, 700-8530 Japan; 2 Department of Pathology, Tianjin Central Hospital of Gynecology Obstetrics, Tianjin, 300100 People’s Republic of China; 3 Laboratory of Nano-Biotechnology, Graduate School of Interdisciplinary Science and Engineering in Health Systems, Okayama University, Okayama, 700-8530 Japan; 4 Department of Microbiology and Biochemistry, Faculty of Pharmacy, Damascus University, Damascus, 10769 Syria; 5 Division of Bioprocess Engineering, Department of Chemical Engineering, Faculty of Engineering, University of Indonesia, Depok, 16424 Indonesia; 6 Division of Biochemistry, Chemistry Department, Faculty of Science, Menoufia University, Shebin El Kom-Menoufia, 32511 Shibin el Kom, Egypt; 7 Okayama University Research Laboratory of Stem Cell Engineering in Detroit, IBio, Wayne State University, Detroit, MI 48202 USA; 8 Department of Pathology, Shanxi Key Laboratory of Carcinogenesis and Translational Research on Esophageal Cancer, Shanxi Medical University, 030001 Taiyuan, PR China; 9 Laboratory of Natural Food & Medicine, Co., Ltd, Okayama University Incubator, Okayama, 700-8530 Japan

**Keywords:** Cancer, Cell biology

## Abstract

Cancer stem cells (CSCs) are a class of cancer cells characterized by self-renewal, differentiation and tumorigenic potential. We previously established a model of CSCs by culturing mouse induced pluripotent stem cells (miPSCs) for four weeks in the presence of a conditioned medium (CM) of cancer cell lines, which functioned as the tumor microenvironment. Based on this methodology of developing CSCs from miPSCs, we assessed the risk of 110 non-mutagenic chemical compounds, most of which are known as inhibitors of cytoplasmic signaling pathways, as potential carcinogens. We treated miPSCs with each compound for one week in the presence of a CM of Lewis lung carcinoma (LLC) cells. However, one-week period was too short for the CM to convert miPSCs into CSCs. Consequently, PDO325901 (MEK inhibitor), CHIR99021 (GSK-3β inhibitor) and Dasatinib (Abl, Src and c-Kit inhibitor) were found to confer miPSCs with the CSC phenotype in one week. The tumor cells that survived exhibited stemness markers, spheroid formation and tumorigenesis in Balb/c nude mice. Hence, we concluded that the three signal inhibitors accelerated the conversion of miPSCs into CSCs. Similarly to our previous study, we found that the PI3K-Akt signaling pathway was upregulated in the CSCs. Herein, we focused on the expression of relative genes after the treatment with these three inhibitors. Our results demonstrated an increased expression of *pik3ca, pik3cb*, *pik3r5* and *pik3r1* genes indicating class IA PI3K as the responsible signaling pathway. Hence, AKT phosphorylation was found to be up-regulated in the obtained CSCs. Inhibition of Erk1/2, tyrosine kinase, and/or GSK-3β was implied to be involved in the enhancement of the PI3K-AKT signaling pathway in the undifferentiated cells, resulting in the sustained stemness, and subsequent conversion of miPSCs into CSCs in the tumor microenvironment.

## Introduction

Alexander A. Maximow, a Russian cell biologist, was the first who proposed the concept of stem cells in 1909^1^. In 1961, the two basic features defining stem cells, namely abilities of self-renewal and differentiation into mature cells, were uncovered by Till and McCulloch^2^. CSCs were first identified in the granulocytes of acute myeloid leukemia in 1994^3^. Since 1997 when Bonnet and Dick identified the CSCs by the ability of self-renewal from a heterogeneous tumor xenograft^4^, Studies have demonstrated that the CSCs exist in various types of cancers, including breast, brain, lung, gastric, colorectal cancers^5^. At present, CSCs represent a unique population of cancer cells that are tumorigenic and resistant to most chemotherapeutic agents and radiation therapy, supporting cancer progression and recurrence.

Recent reports have described that the plasticity of non-CSCs plays a crucial role in promoting the dynamic conversion of non-CSCs into CSCs, which exhibit hierarchical heterogeneity^6–8^. Although the factors involved in regulating the conversion have not been identified, epithelial-mesenchymal transition is closely related to the presence of CSCs. The phosphoinositide 3-kinase (PI3K), protein kinase B (AKT) and mammalian target of rapamycin (mTOR), which constitutes the core cascade called the PI3K/AKT/mTOR signaling cascade, have regulatory roles in cell survival, proliferation, and differentiation, and a critical role in tumorigenesis^9^. PI3K is reported to be associated with the development of various human tumors, including breast cancer, lung cancer, melanoma and lymphomas^10–14^. Furthermore, the PI3K downstream kinase, AKT, is reported to be involved in malignant transformation^15^. In this context, some environmental factors could be important stimulants to understand the mechanism of conversion of non-CSCs to CSCs.

Some chemical compounds are well known as mutagens and/or carcinogens. Some as inhibitors of cytoplasmic signaling pathways and others as both mutagens and/or carcinogens and inhibitors. Chemical compounds are usually evaluated for their risk to induce cancer by various assays such as mutagenicity assays, repeated dose toxicity studies, and statistical analyses.

We have previously been investigating the new insight in the CSCs by developing a CSC model that is derived from iPSCs, which were reprogrammed from normal cells. CSCs are now widely considered as a small population of cells present in malignant tumors exhibiting self-renewal and differentiation potential responsible for the malignancy. However, the mechanism of the appearance of CSCs is not well explained yet. Considering the microenvironment is crucial to regulate the proliferation, self-renewal potential and differentiation of normal stem cells or progenitor cells in each tissue, imbalanced abnormal microenvironment such as chronic inflammation lasting a long time affecting on the plastic stem cells could be the stimulant to convert the normal stem cells into CSCs. In this context, microenvironment distorted by the factors secreted from cancer cells could affect the diverse directions of stem cells and lead to the characteristics of CSCs. Even though a CSC shares with normal stem cells in the characteristics of maintaining the stemness and differentiation potential, multiple genetic and epigenetic regulations are different to acquire the features of CSCs. The conversion of miPSCs into CSCs was demonstrated by the treatment with a CM prepared from different cancer cell lines, for a period of four weeks^16–18^. This implies that the essential factors should be contained in the CM derived from cancer cells mimicking the tumor microenvironment. In the current study, we proposed a simple method to assess the risks of tumor-inducing factors in the presence of chemical compounds that accelerated the conversion of miPSCs into CSCs. Furthermore, we used the signaling inhibitors to elucidate the signaling pathways responsible for the appearance of CSCs.

## Materials and Methods

### Materials

miPSCs, (iPS-MEF-Ng-20D-17; Lot No. 012) were provided by the RIKEN Cell Bank, Japan. DMEM, fetal bovine serum (FBS), 2-mercaptoethanol, collagenase, gelatin, Hematoxylin and Eosin Y were purchased from Sigma, NY. EBM-2 media Endothelial cell basal medium-2 and EBM-2 SingleQuots Kit were from Lonza, MD, USA. Matrigel was from Corning Inc., Corning, NY, USA. KnockOut Serum Replacement (KSR) and Non-Essential Amino acids (NEAA) were from Gibco, NY. L-Glutamine, 2.5 % Trypsin and CaCl_2_ were from Nacalai Tesque, Japan. Paraformaldehyde, Hank’s balanced salt solution (HBSS) and 100 U/ml penicillin/streptomycin (P/S) cocktail were from Wako, Japan. Leukemia Inhibitory Factor (LIF) was from Millipore, MA. Mitomycin C treated mouse embryonic fibroblasts (MEFs) were provided by Reprocell, Japan. Lewis lung carcinoma (LLC) cells were from ATCC, VA, USA. Insulin-transferrin-selenium-X (ITS-X) was from Life Technologies, CA. Signal transduction inhibitors used in this study were obtained as listed in Table S2.

Anti-mouse Ki67 rabbit polyclonal antibody (ab15580), anti-mouse Sox2 rabbit polyclonal antibody (ab97959) and anti-mouse panCK rabbit polyclonal antibody (ab191208) were purchased from Abcam, UK. Anti-mouse Erk1/2 (p44/42 MAPK) rabbit polyclonal antibody (4695), anti-mouse p-Erk1/2 (Thr202/Tyr204) rabbit polyclonal antibody (4370), anti-mouse Akt rabbit polyclonal antibody (9272), anti-mouse pAkt (Ser^473^) rabbit polyclonal antibody (9271), anti-mouse β-catenin rabbit polyclonal antibody (9562) , anti-mouse β-actin rabbit polyclonal antibody (4970), horseradish peroxidase (HRP)-conjugated anti-rabbit IgG goat polyclonal antibody (7074) or anti-mouse IgG goat polyclonal antibody (7076) were purchased from Cell Signaling Technology, MA, USA.

### Cell culture

The miPSCs were designed to express GFP gene under the control of Nanog promoter, so that the undifferentiated state is easy to distinguished from differentiated state^19^. The miPSCs were maintained in DMEM supplemented with 15% FBS, 0.1 mM NEAA, 2 mM L-Glutamine, 50 U/ml P/S, 0.1 mM 2-mercaptoethanol and 1000 U/ml of LIF on feeder layers of mitomycin C treated MEF. In the case of feeder-free, miPSCs were cultured on gelatin-coated dishes. LLC cells were maintained in DMEM containing 10% FBS supplemented with 100 U/ml P/S.

For primary cultured cells, the mouse allografts were excised and cut into small pieces (approximately one mm^3^), then washed three times by HBSS and transferred them into a 15-ml tube contained with 4 ml of dissociation buffer, which was prepared as PBS containing 0.25% trypsin, 0.1% collagenase, 20% KSR, 1 mM of CaCl_2_, then incubated at 37 °C for 40 mins. Adding 5 ml of DMEM containing 10% FBS to terminate the digestion. The suspension of cells was transferred into the new tubes, centrifuged at 1000 rpm for five mins. The cell pellet was re-suspended in 5 ml HBSS, and centrifuged at 1000 rpm for five mins, then placed into an appropriate volume of miPS medium without LIF and seeded the cells into a dish at a density of 5 × 10^5^/ml. For removing the host cells in primary cells should be treated with puromycin for 24 h.

To prepare the CM from LLC cells, the medium was collected as previously described^16^. The mixture of CM and miPS medium (1:1) was supplemented with each signal inhibitor (Table S2). miPSCs were cultured in the mixed medium without LIF and MEF feeder cells. One half of the medium was replaced with fresh mixed medium every day, up to day 6. On day 7, the expression of green fluorescent protein (GFP) and cell morphology was observed, and photographed using an inverted fluorescent microscope (Olympus IX81, Japan).

### Green fluorescent protein (GFP) Assay

miPSCs cells were seeded at a density of 2 × 10^3^ cells/well in 96-well black plates (Corning Inc., NY) using miPS medium overnight. On the second day, half of the medium was replaced with a fresh medium mixed with compounds every day, up to day six. On day seven, the cells were washed twice with 1× PBS and lysed in a 50 μl/well lysis buffer followed by the incubation at 37 °C for 5mins. The relative fluorescence intensity was then measured at 509 nm, excited at 488nm, using a microplate reader (SH-9000lab, Corona, Japan). The read of each well was obtained from 9 points at a distance of 1 mm from the bottom of each well flashed 30 times.

The GFP fluorescence from the miPSCs cultured in CM without signal inhibitors was used as a control to determine the threshold of the bottom line in order to distinguish the effect of the signal inhibitors. The fluorescence from 6 wells was measured 5 times from independent experiments, the averages and standard deviations of the readings were calculated (Table S1). Next, we assessed the effect of the inhibitors in concentrations between 0 and 10 μM by the intensity of the GFP fluorescence and determined the optimal concentration which enhanced the fluorescence of GFP (Table S2).

### Sphere formation assay

The cells were seeded at a density of 1 × 10^4^ cells/well in 24-well ultra-low attachment plates (Corning Inc., NY) in serum-free medium consisting of 97.5% DMEM, 0.5 U/ml P/S, 0.1 mM NEAA, 1 mM L-Glutamine, 0.1 mM 2-mercaptoethanol and 1% v/v ITS-X. After 5 to 7 days, the number of spheroids was counted, and images were captured using an inverted microscope (CKX41, Olympus, Japan) or an inverted fluorescent microscope (IX81, Olympus, Japan).

### Tube formation assay

The cells were seeded at a density of 5 × 10^5^ cells/60 mm dish and incubated at 37 °C with 5% CO_2_. When the formation of a 70% confluent monolayer, collected and re-suspended 5 × 10^5^ cells in EBM-2 media and seeded in 12-well plates, coated with growth factors reduced Matrigel for 24h in the presence of a different growth factors, such as human epidermal growth factor (EGF, 5 ng/mL), human vascular endothelial growth factor (VEGF, 0.5 ng/mL), R3-insulin-like growth factor-1 (R3-IGF-1, 20 ng/mL), ascorbic acid (1 μg/mL), hydrocortisone (0.2 μg/mL), human basic fibroblast growth factor (bFGF, 10 ng/mL), heparin (22.5 μg/mL) and 2% FBS. Each experiment was performed in triplicate, and the images were captured by using an Olympus IX81 microscope (Olympus, Tokyo, Japan).

### Animal experiments

The animal experiments were reviewed and approved by the ethics committee for animal experiments at the Okayama University under the ID OKU-2016078. All experiments were performed according to the policy on the care and use of the laboratory animals, Okayama University. Thirty nude mice (Balb/c-nu/nu, female, four weeks) were purchased from Charles River (Kanagawa, Japan) and kept at 23 °C and fed with sterilized food and water during the experiments. Cells at 1 × 10^6^ were suspended in 200 μl of HBSS and were subcutaneously transplanted into each mouse. Three mice were used for each inhibitor.

At the end of the experiments, the mice were euthanized by the isoflurane-euthanasia method (Laboratory Animal Anaesthesia (3rd Ed.) Paul A. Flecknell 2009). Five percent of isoflurane (DS Pharma Animal Health, Japan) was exposed to the mice and the exposure was continued until one minute after their breathing stopped. Finally, euthanasia was confirmed by cervical dislocation.

### RNA extraction, cDNA synthesis and reverse transcription quantitative PCR analysis

Total RNA was extracted from cells with the RNAeasy Mini kit (QIAGEN, Germany) and one μg of RNA was reverse transcribed by the superscript first-strand kit (Invitrogen, CA). Reverse transcription-quantitative PCR (RT-qPCR) was performed with LightCycler 480 SYBR Green I Master Mix (Roche, Switzerland). The primers were described in Table S3.

### Histological analysis and immunohistochemistry

For histological analysis, tumors were excised from the mice 4–6 weeks after transplantation. The paraffin-embedded tissue samples were cut into 5 μm-thickness sections, deparaffinized and stained with 0.5% Hematoxylin and Eosin Y (HE). The primary antibodies and dilutions used for IHC were as follows: 1:200 of anti-Ki67 antibody, 1:200 of anti-Sox2 antibody and 1:200 of anti-panCK antibody.

### Western blotting

Proteins extracted from the miPSCs treated with each condition were subjected to SDS-PAGE, transferred to Immobilon-FL transfer membrane (PVDF, Merck Millipore, Germany) and probed with antibodies against Akt at a dilution of 1:1000, pAkt (Ser^473^) at a dilution of 1:1000, Erk1/2 at a dilution of 1:1000, p-Erk1/2 (Thr202/Tyr204), at a dilution of 1:1000, β-catenin at a dilution of 1:1000 and β-actin at 1:1000. This was followed by the secondary antibody, either HRP–conjugated anti-rabbit or anti-mouse IgG at dilutions of 1:2000 or 1:5000, respectively.

### Flow cytometry

miPSCs that survived following one week of treatment were transferred to a 60-mm dish and harvested during the logarithmic growth phase. Cells were re-suspended in 100 μl of ice-cold PBS and analyzed by a flow cytometer (BD Accuri C6 plus, Becton & Dickinson, NJ). Data from each experiment was analyzed by FlowJo software (FlowJo, LLC, Ashland, OR, USA).

### Statistical analysis

The data were analyzed by the two-tailed student’s t-test using the Prism Software version7 (Graph Pad Software, San Diego, CA, USA). Results are presented as the mean ± standard deviation (SD) from independent experiments. Each experiment was performed in triplicate. P < 0.05 was statistically significant.

### Ethics approval and consent to participate

The plan of animal experiments was reviewed and approved by the ethics committee for animal experiments of Okayama University under the ID: OKU-2016078 (2016).

## Results

### Evaluation of the effect of chemical compounds on the survival of miPSCs

**Figure 1:**
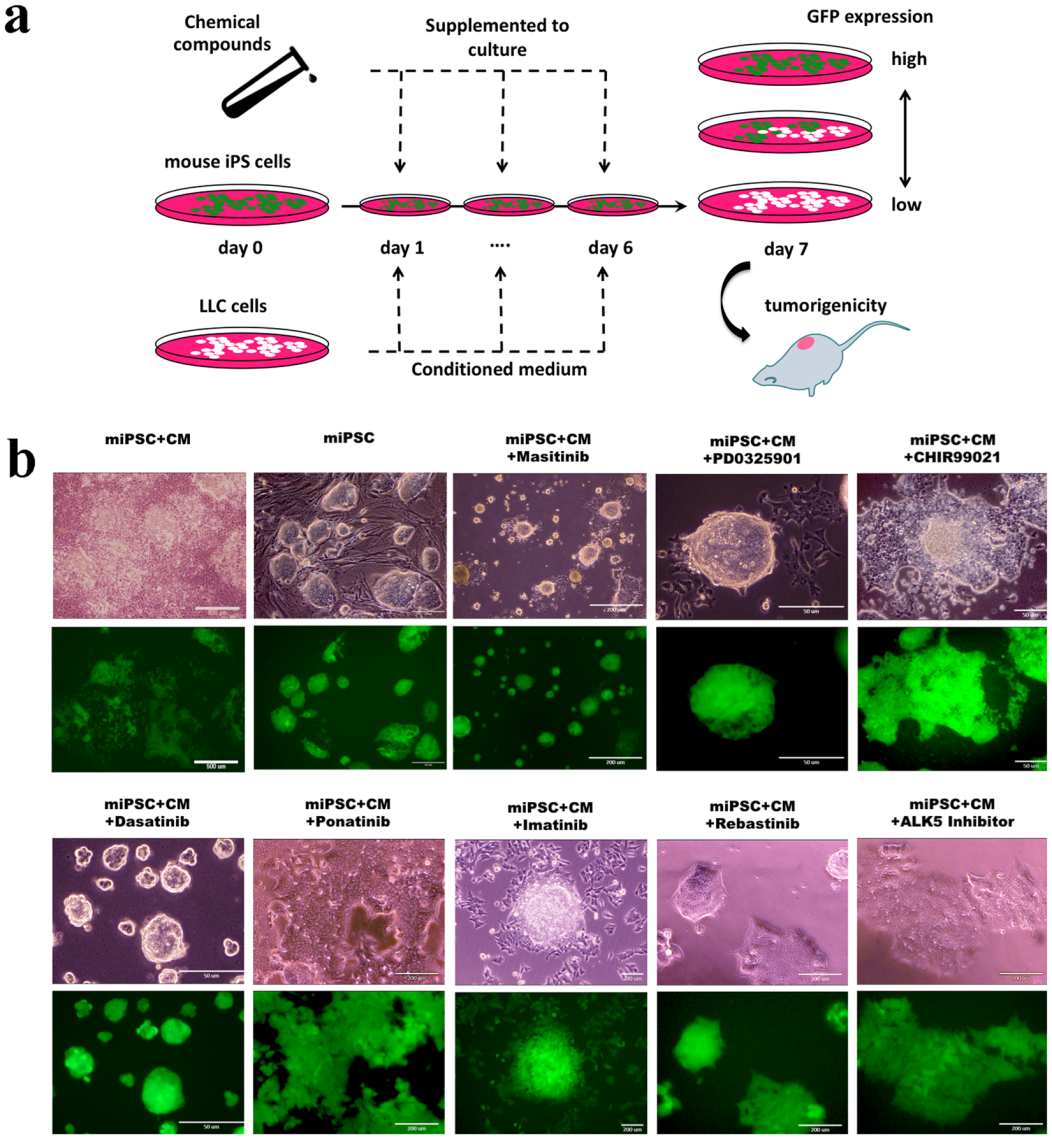
The conversion of miPSCs into CSCs by the treatments with chemical compounds. (**a**) Schematic flow chart of the conversion from miPSCs into CSCs by chemical compounds. (**b**) Representative images of the conversion from miPSCs into CSCs. Cells were cultured with media containing CM and chemical compounds, and colonies were observed for the GFP expression after 1-week treatment.

Previously, we have described the development of CSCs from miPSCs using CM from cancer cell lines, during a 4 week or longer period^16–18^. Based on these previous experiments, we treated the miPSCs with each compound for 1 week in a CM of Lewis lung carcinoma (LLC) cells. However, 1 week was too short a period for the CM to induce the conversion of the miPSCs into CSCs (Fig. 1a). We postulated that the short time required to assess the development of CSCs from miPSCs could be utilized to evaluate non-mutagenic chemical compounds mediating the conversion of miPSCs to CSCs. To establish this procedure, we adopted miPSCs expressing a gene encoding the green fluorescent protein (GFP) under the control of *Nanog* promoter^16^ to monitor the potential of stemness by green fluorescence under a microscope or using a microplate reader. This procedure roughly distinguished the conditions that enhanced the survival of miPSCs, including the conversion into CSCs, by the expression of GFP fluorescence. Following a 1-week treatment, the GFP fluorescence of miPSCs persisted during the undifferentiated state, while GFP fluorescence decreased when the miPSCs normally differentiated to die in the absence of Leukemia Inhibitory Factor (LIF). We evaluated the effect of compounds in the conversion of miPSCs into CSCs in the presence of a CM derived from LLC cells. We found certain compounds enhanced the expression of GFP in miPSCs while some decreased, and others gave no significant effect (Fig. 1b). Subsequently, we evaluated each of the 110 candidate compounds to establish the threshold and optimal concentration by detecting the intensity of GFP fluorescence (Tables S1 and S2).

### Stemness and differentiation of GFP positive miPSCs after 1-week treatment

**Figure 2:**
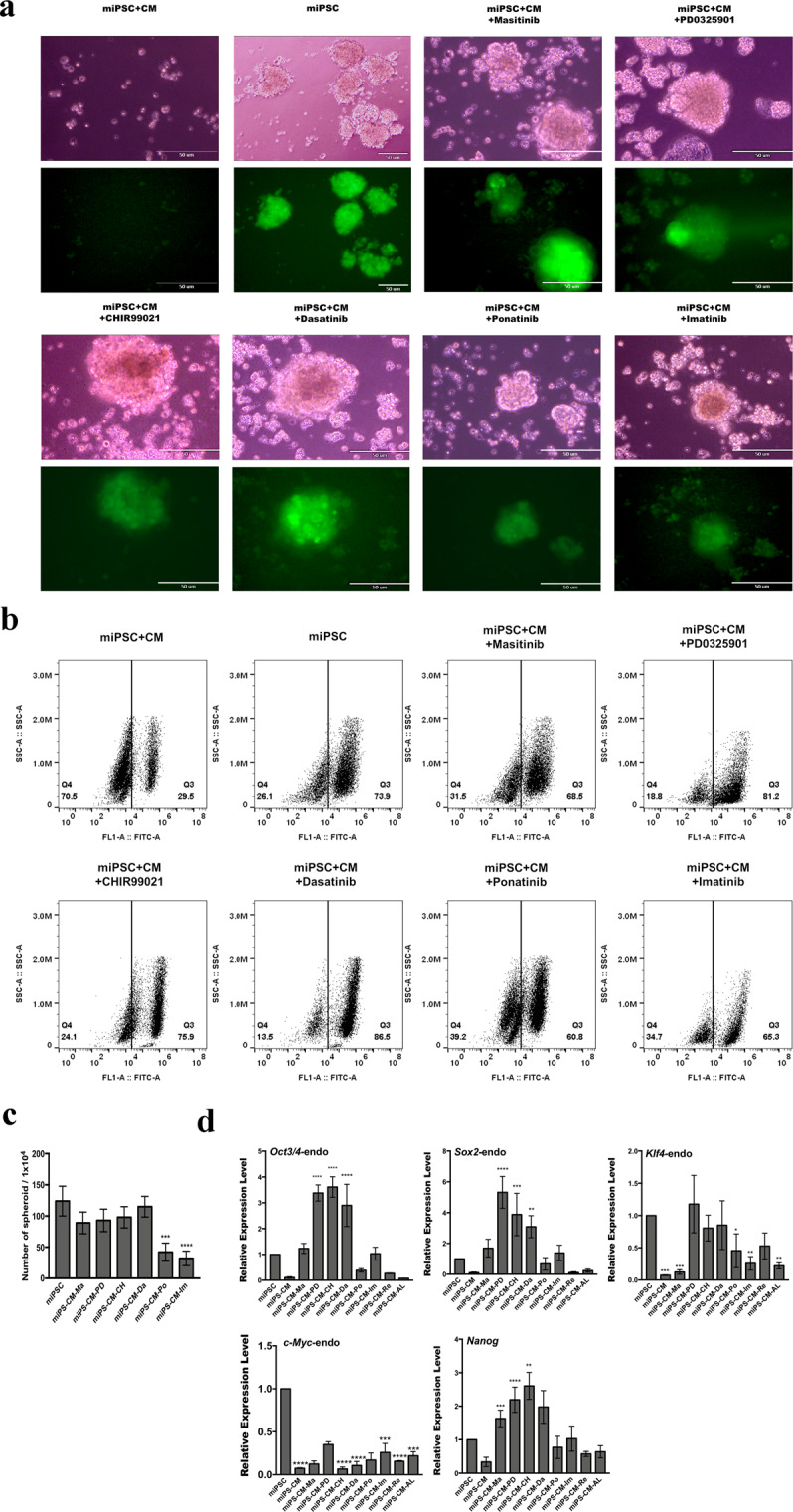
Positive chemical compounds promote self-renewal capacity in the conversion of miPSCs into CSCs. (**a**) Sphere formation assay shows spherogenic potential and the expression of GFP. (**b**) Graphical representation of the number of spheroids after the conversion of 1-week. (**c**) Flow cytometric analysis shows GFP positive population in the conversion cells after treatment with chemical compounds. (**d**) The expression levels of stemness markers (endogenous genes) were analyzed by RT-qPCR. The data were analyzed using ordinary one-way ANOVA multiple comparisons and is presented as the mean ± standard deviation ****P < 0.0001, ***P < 0.001, **P < 0.01, *P < 0.05.

Previous evidence suggests that the ability to form spheroids is associated with CSCs properties^20^. GFP-positive cells that survived following one week of treatment were further assessed for the sphere-forming potential. We assessed the chemical compounds, which exhibited positive capability to the cell survival following 1-week treatment (Table S2), for sphere formation potential in suspension culture. Our results indicated that six compounds promoted sphere formation (Fig. 2a,b), while the remaining candidate compounds failed to demonstrate this property. The surviving cells were then cultured under adhesive conditions and analyzed by flow cytometry, with approximately 60–80% of the cells expressing GFP (Fig. 2c).

We assessed the expression of endogenous stemness markers *Oct3/4, Sox2, Nanog*, *Klf4* and *c-Myc*, which have a dominant role in the maintenance of embryonic stem cells (ESCs) and iPSCs, and self-renewal in the surviving cells, using RT-qPCR. The expression level of each endogenous gene and transgene was confirmed by using specific primers^21^. The expression of stemness markers, Oct3/4, Sox2 and Nanog, was significantly upregulated in miPSCs when treated with PD0325901, CHIR99021 and Dasatinib (Fig. 2d). However, the cells treated with Masitinib did not show significance difference in the expression of both oct3/4 and sox2 but only Nanog was upregulated. As for the expression of Klf4 gene, PD0325901, CHIR99021 and Dasatinib maintained almost at the same level of miPSCs while Masitinib decreased the expression. That of c-Myc gene was suppressed in every treatment. We did not detect any aberrant activation of the transgene, pertinent to viral-transduction for the establishment of miPSC. Since PD0325901, CHIR99021 and Dasatinib feasibly enhanced the sphere formation and the expression of the stemness marker. The amount of *Nanog* and *Oct3/4* increased in miPSCs when treated with these three compounds. Collectively, PD0325901, CHIR99021 and Dasatinib were confirmed to maintain the stemness of miPSCs and the resultant cells were designated as miPS-LLCcm-PD, miPS-LLCcm-CH and miPS-LLCcm-Da, respectively.

The differentiation potential of the treated cells was evaluated by tube formation exhibiting the phenotype of endothelial cells (Fig. S1). Also, miPSCs were cultured in adhesive condition without LIF (Fig. S2). As the result of differentiation, cells lost GFP expression after 3 days and died after 1 week due to cell senescence.

### Tumorigenicity of GFP positive miPSCs after 1-week treatment

**Figure 3:**
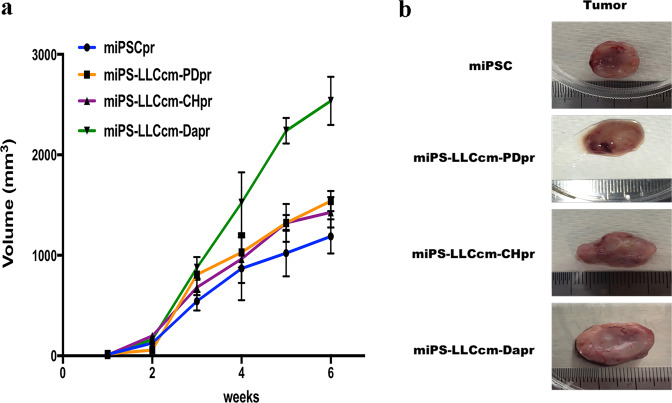
Tumorigenicity of CSCs converted from miPSCs treated with chemical compounds. (**a**) The tumor growth in 6 weeks. (**b**) Generation of tumors after subcutaneous transplantation of miPSCs and the 1 week of converted cells.

**Figure 4:**
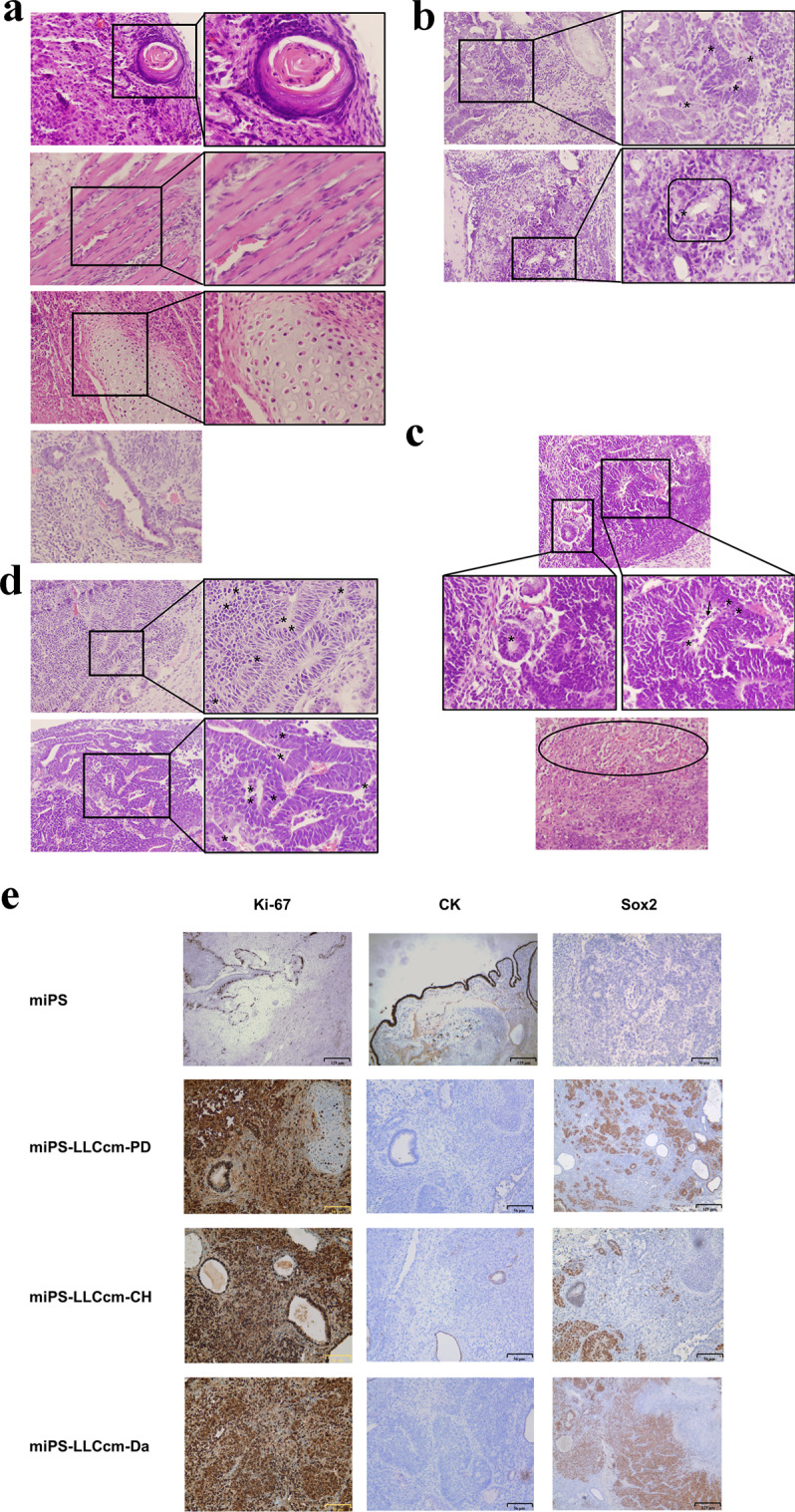
Histopathological observation of the tumors formed by the CSCs. Benign teratoma formed by miPSCs. (**a**) transplanted subcutaneously. Normal tissue types derived from three germ layers, including squamous epithelium (keratinized ball), skeletal muscle, cartilage and benign glandular epithelium are observed. Sections from the tumors formed by miPS-LLCcm-PD (**b**) miPS-LLCcm-CH (**c**) and miPS-LLCcm-Da (**d**) cells transplanted subcutaneously. Malignant structures are observed in the glandular cavities (square, bottom right in b) composed of multiple abnormal glands, which are crowded back to back exhibiting high nuclear to cytoplasmic ratio, severe nuclear atypia and multiple pathological mitotic figures (asterisks in **b**–**d**). Abnormal glands, inside of glandular cavity has necrosis (arrow in **c**), and large area necrosis (oval in c). Original magnification was 20X and 40X (**a**–**d**). (**e**) Immunohistochemical analysis showed malignancies with highly proliferative areas strongly stained for Ki-67 and poorly differentiated areas, poorly stained for CK and strongly for Sox2 in the tumor formed by miPS-LLCcm-PD, miPS-LLCcm-CH and miPS-LLCcm-Da when compared with the benign teratoma formed by miPSCs.

Considering the self-renewal capacity of these cells, and the results from our previous study, we investigated whether these compounds were effective in promoting the conversion from miPSCs into CSCs. To evaluate the tumorigenicity of these cells, 1 × 10^6^ of these cells were subcutaneously transplanted into Balb/c nude mice, and the tumor formation was monitored for 6 weeks (Fig. 3a). Tumors formed were excised at 6-weeks (Fig. 3b) and subjected to histological and immunohistochemical analysis (Fig. 4). As previously reported^16^, miPSCs formed a teratoma phenotype with various normal germ layers, including the squamous epithelium (keratinized ball), skeletal muscle, cartilage and benign glandular epithelium (Fig. 4a). Tumors derived from miPSCs treated with PD0325901, CHIR99021 and Dasatinib formed malignant tumors, sections of which demonstrated poorly differentiated phenotype, high nuclear to cytoplasmic ratio, severe nuclear atypia and multiple pathological mitotic figures (Fig. 4b–d). They also exhibited multiple abnormal glands, trabecular patterns, necrosis in the glandular cavity, and large zones of necrosis in the mesenchymal tissue. Immunohistochemical analysis demonstrated that the staining with Ki-67 antibody was highly proliferative, supporting the rapid tumor growth. The expression of CK and Sox2 indicated phenotypes of poor differentiation (Fig. 4e). Hence, the development of malignant tumors was confirmed by the GFP positive miPSCs treated with the inhibitors for one week (Table 1).

**Table 1:** Summary of tumorigenic potential of miPSCs treated in various conditions.

Supplement	Conditioned medium	Cell number	Tumor formation	Histologic examination
LIF (1000U/mL)	−	1 × 10^6^	3/3	Benign teratoma
−	+	1 × 10^6^	0/3	−
Mastinib (6.25 μM)	+	1 × 10^6^	0/3	−
PD0325901 (5 μM)	+	1 × 10^6^	3/3	Malignant tumor, adenocarcinoma
CHIR99021 (2.5 μM)	+	1 × 10^6^	3/3	Malignant tumor, adenocarcinoma
Rabastinib (2.5 μM)	+	1 × 10^6^	0/3	−
ALK5 Inhibitor (10 μM)	+	1 × 10^6^	0/3	−
Dasatinib (1.25 μM)	+	1 × 10^6^	3/3	Malignant tumor, adenocarcinoma
Imatinib (2.5 μM)	+	1 × 10^6^	0/3	−
Ponatinib (0.625 μM)	+	1 × 10^6^	0/3	−

### Self-renewal and stemness capacities of primary cells derived from tumors

**Figure 5:**
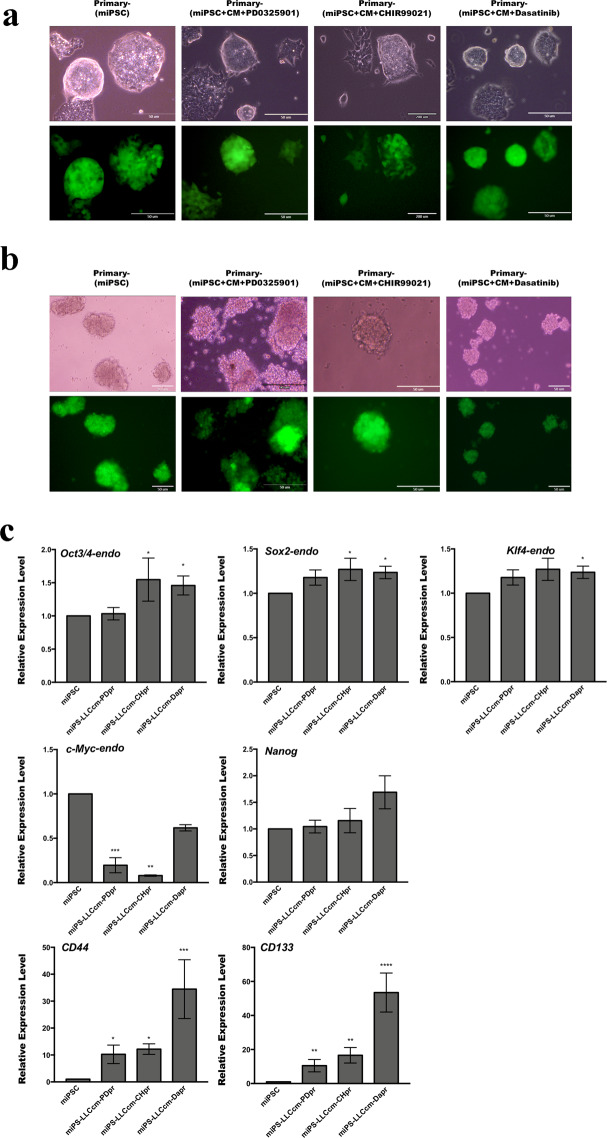
Primary culture cells possess self-renewal capacity. (**a**,**b**) Primary cells in adherent culture and sphere formation in suspension culture with the expression of GFP. (**c**) RT-qPCR analysis of stemness markers in primary tumor cells. The data were analyzed using ordinary one-way ANOVA multiple comparisons and presented as the mean ± standard deviation ****P < 0.0001, ***P < 0.001, **P < 0.01, *P < 0.05.

To further evaluate the properties of primary tumors, the primary cells were cultured from the tumors developed in the previous section. The adhesive culture of the primary cells was positive for GFP, indicating that they originated from the miPSCs (Fig. 5a). In suspension culture, these cells were able to form spheroids indicating the self-renewal capacity, and all spheroid-forming cells expressed GFP (Fig. 5b). The expression levels of *Oct3/4*, *Sox2*, *Nanog*, and *Klf4* were relatively maintained or slightly enhanced in the primary cells when compared with miPSCs (Fig. 5c). However, the expression of *c-Myc* was decreased, suggesting a negligible contribution of this gene to the conversion of miPSC to CSC. Therefore, endogenous *Oct3/4*, *Nanog*, *Sox2* and *Klf4* might contribute to these properties. We further evaluated the expression of CSC markers, CD44 and CD133 in primary cultured cells. These markers found increased in treated cells when compared with those in miPSCs.

### Signaling inhibitors accelerated the conversion of miPSCs into CSCs activating the PI3K-AKT pathway

**Figure 6:**
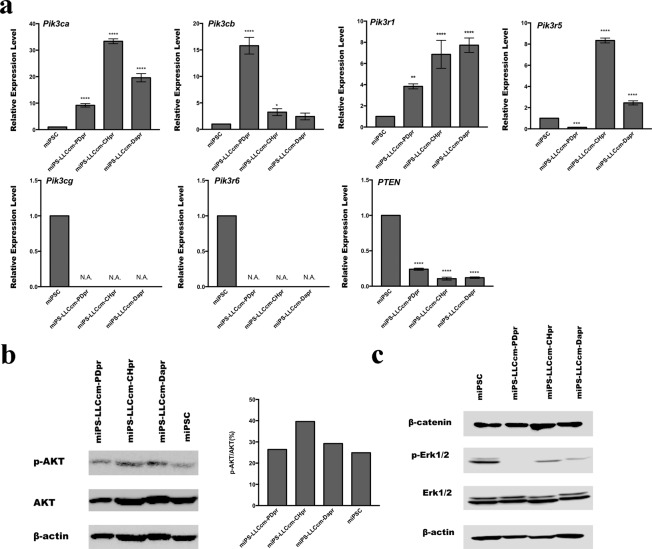
PI3K signaling pathway was activated in primary culture cells. (**a**) rt-qPCR analysis of *Pik3ca*, *Pik3cb*, *Pik3cg, Pik3r1*, *Pik3r5, Pik3r6* and *PTEN* expression. (**b**) Western blotting analysis of the AKT expression and phosphorylation. (**c**) Western blotting analysis of the β-catenin, Erk1/2 and phosphorylated Erk1/2.

The primary cultured cells obtained from the malignant tumors developed by transplanting miPSCs treated with PD0325901, CHIR99021 and Dasatinib were named as miPS-LLCcm-PDpr, miPS-LLCcm-CHpr, miPS-LLCcm-Dapr, respectively. The signaling pathways of Ras-Raf-MEK-ERK, Abl, Src, c-Kit, Wnt-GSK3 and PI3K-Akt-mTOR are considered typically important in cancer cell growth and survival^22–24^. Our results demonstrated that the inhibition of the pathways, expect PI3K, was effective in enhancing the induction of the CSCs. On the other hand, PI3K inhibitors did not enhance the induction of CSCs (Table S2). Furthermore, in our previous study, we reported that PI3K-AKT signaling pathway was up-regulated in the CSCs developed from miPSCs^25^. Therefore, we evaluated the expression of PI3Ks in the CSCs, developed from miPSCs, in the presence of PD0325901, CHIR99021 and Dasatinib. Simultaneously, the expression of PTEN, which is considered to be antagonized by PI3K through the dephosphorylation of PIP_3_^26^, was evaluated. We assessed the expression of *Pik3ca*, *Pik3cb*, *Pik3cg*, *Pik3r1*, *Pik3r5*, *Pik3r6* and *PTEN* using RT-qPCR in the primary cultured cells (Fig. 6a). In the comparison to the miPSCs, *Pik3ca*, *Pik3cb*, *Pik3r1* and *Pik3r5* showed significantly higher expression in the miPS-LLCcm-PDpr, miPS-LLCcm-CHpr and miPS-LLCcm-Dapr cells while *PTEN* showed lower expression, and *Pik3cg* and *Pik3r6* demonstrated no expression. We then confirmed the expression of AKT1, AKT2 and mTOR using RT-qPCR (Fig. S3). We assessed the phosphorylation of AKT by western blotting (Fig. 6b) and found that the phosphorylation was enhanced in miPS-LLCcm-PDpr, miPS-LLCcm-CHpr, miPS-LLCcm-Dapr cells when compared with miPSCs. The western blotting of β-catenin, Erk1/2 and phosphorylated Erk1/2 showed inhibition or decrease of phosphorylation of Erk1/2 in the cells treated with chemicals while the amount of β-catenin was not apparently affected (Fig. 6c). These findings are consistent with the enhanced PI3K-AKT signaling pathway in CSCs, as demonstrated in our previous study^25^.

## Discussion

The carcinogenic risk assessment of chemical compounds has always been performed by different procedures such as mutagen tests, repeated dose toxicity studies, and statistical analyses. Since these procedures are considered to involve oncogenic mutations, it takes a long time to assess the risks. However, few rapid methods to evaluate the carcinogenic risk of non-mutagenic chemical compounds are found. In this study, we proposed a simple and feasible method for preliminary screening of carcinogens. Based on the previous reports on the induction of CSCs^16–18^, we exploited miPSCs expressing GFP under the control of the *Nanog* promoter^19^. It is worthwhile noticing that the miPSCs were reprogrammed from normal embryonic fibroblasts, but not from cancer-derived cells. Since *Nanog* was considered as a marker widely associated with stemness^27,28^, the assessment procedure was designed to observe GFP in the miPSCs under TME for 1-week, which was not long enough to induce sufficient oncogenic mutations, in the presence of each chemical compound. One hundred and ten non-mutagenic chemical compounds, most of which were known as inhibitors of cytoplasmic signaling pathways, were applied on the procedure to assess their risk of inducing CSCs. Twenty out of 110 compounds exhibited GFP fluorescence significantly maintained or enhanced in miPSCs. After evaluating the colony-forming efficiency, we selected eight chemical compounds. Further, sphere formation, which contributes to self-renewal to maintain stemness, was successful to select six out of these eight compounds. Finally, three compounds, PD0325901, CHIR99021 and Dasatinib, were found to facilitate tumor formation *in vivo*. The resultant cells exhibit the spheroid formation in the suspension culture and high tumorigenic potential as the basic characteristics of CSCs^29–31^, so we concluded that miPSCs were converted into CSCs. Although PD0325901, CHIR99021 and Dasatinib are considered to suppress the growth of cancer cells as anti-cancer agents, we found in this study these compounds might be inducing CSCs under the TME. These results could help find some mechanisms to explain the resistance of CSCs against chemotherapy in the future.

Dasatinib is a known tyrosine kinase inhibitor of ABL1/BCR-ABL1, whereas Masatinib as c-Kit. Interestingly, miPSCs treated with Masatinib were not tumorigenic and Masatinib was not indicated as an inducer of CSCs in this study. As c-Kit is known as the Stem Cell Factor receptor, Masatinib could have inhibited the signaling pathway essential for the self-renewal. On the other hand, Dasatinib might inhibit the growth of certain cells at partially differentiated stages, resulting in the enhancement of self-renewal of the undifferentiated cells. These results may imply the different functions of tyrosine kinases involved in the stages of cellular differentiation.

PD0325901 is well known as a MEK1/2 inhibitor. MEK is a main downstream tyrosine kinase from the Ras/Raf/MAP kinase cascade^32^, and PD0325901 appeared to conceivably induce the conversion of miPSCs to CSCs, as Dasatinib did.

CHIR99021 is an inhibitor of GSK-3β, which plays a role in the downstream signaling of Wnt. As Wnt suppresses the function of GSK-3β, CHIR99021 could act as Wnt resulting in the activation of β-catenin-Lef/Tcf signaling, considered necessary to maintain CSCs^33–35^. Ying *et al*. reported that the self-renewal potential of mouse ESCs was maintained in the presence of CHIR99021 and PD0325901^36^. In this context, down-modulation of GSK-3β could result in a crucial stage to maintain metabolic activity, biosynthetic capacity, and overall viability, resulting in the decrease of phospho-ERK through the attenuation of MEK1/2, which reversed, would support our results. Hence, these inhibitors appear to relatively activate the PI3K/AKT pathway.

Recently, we have found the overexpression of *pik3r5* and *pik3cg*, components of class IB PI3 kinase, in CSCs derived from miPSCs cultured in the presence of CM^25^. We could postulate again that the PI3K/AKT pathway might be activated in miPS-LLCcm-PD, miPS-LLCcm-CH, miPS-LLCcm-Da cells, which were resulted from the 1-week treatment in this study. The western blotting analysis detected a significant amount of immunoreactive PI3K and phosphorylated AKT in the primary cultures derived from transplanted tumors (Fig. 6b). In accordance with the results obtained, we further investigated the expression levels of each moiety of PI3K, namely *Pik3ca*, *Pik3cb*, *Pik3cg*, *Pik3r1*, *Pik3r5* and *Pik3r6*. We found the overexpression of *Pik3ca*, *Pik3cb, Pik3r1* and *Pik3r5* as the candidates for further evaluation. *Pik3r1*and *Pik3ca*/*Pik3cb* are the components of class IA PI3K while *Pik3r5*, a moiety of class IB, was found in our previous report^25^. The expression of *Pik3cg*, the half moiety of class IB for *Pik3r5*, was too low to be amplified by RT-qPCR, and hence class IB PI3K was not considered as active. Moreover, the combination of *Pik3r1* and *Pik3ca* formed PI3K that is activated through tyrosine kinase receptors and *Pik3r1* and *Pik3cb* formed PI3K that is activated by receptor tyrosine kinases and/or G Protein-Coupled Receptors (GPCRs). Therefore, the resultant CSCs could acquire a variety of potential critical features in mediating several aspects of cellular function, including nutrient uptake, metabolic reactions, cell growth and survival through PI3K/AKT signaling pathways in response to various ligand stimulation. This analogy may coincide with the several previous studies demonstrating PI3K and AKT were frequently hyper-activated in a majority of cancers^37,38^ while the mechanisms by which PI3K regulated self-renewal and pluripotency, remain somewhat elusive. However, the contribution of PI3K/AKT signaling in preserving the ability of iPSCs to self-renewal and differentiation has been delineated in recent studies^39,40^.

Hishida *et al*. suggested that PI3K promoted the retention of ES cell properties mainly through the inhibition of the two downstream pathways, the mitogen-activated protein kinases/extracellular signal-regulated kinase (MAPK/Erk) pathway and the GSK3 signaling pathway^33^. It is worthwhile noticing that the phosphorylation of Erk was inhibited by the treatment with CHIR99021 (GSK-3β inhibitor) and Dasatinib (Abl, Src and c-Kit inhibitor) as well as PDO325901 (MEK inhibitor) due to probably by the crosstalk of the signaling (Fig. 6c). This observation could be supported by the down-regulation of *c-Myc* (Fig. 2d), which coincided with the results reported by Maranpon *et al*.^41^. Simultaneously, the effect of GSK-3β inhibitor was not apparent on the amount of β-catenin as found in Fig. 6c probably because all the cells were maintaining the stemness, which was considered to be supported by the Wnt signaling. The crosstalk mechanism in this point should be further investigated in the future. Previous studies from several groups have demonstrated that certain AKT downstream factors, such as *Oct3/4*, *Sox2* and *Nanog*, played an important role in the self-renewal of ESCs and the early development of embryos^42–44^. Meanwhile, these markers were also found as the targets of the AKT signaling pathway phosphorylating T^235^ in *Oct4*, to maintain stemness and inhibit the differentiation of iPSCs^45^. The enhanced conversion of the iPSCs into CSCs could be explained similarly.

## Conclusions

miPSCs expressing the GFP gene under the control of the *Nanog* promoter were exploited to screen the carcinogens in 1 week under TME. As the results showed, PDO325901, CHIR99021 and Dasatinib were found to enhance the induction of CSCs. Taking the signaling pathways inhibited by these compounds into consideration, the inhibition of Erk1/2, tyrosine kinase and/or GSK-3β were implied to be involved in the enhancement of the PI3K-AKT signaling pathway, resulting in the sustained stemness and exerting tumorigenic potential in miPSCs under TME.

## Supplementary information

Supplementary Figure S1.

Supplementary Figure S2.

Supplementary Figure S3.

Supplementary Information.

Supplementary Table S1.

Supplementary Table S2.

Supplementary Table S3.

## Data Availability

All data generated or analyzed during this study are included in this published article and its Supplementary File.
