# Supplementary figures and images for: Signaling Inhibitors Accelerate the Conversion of mouse iPS Cells into Cancer Stem Cells in the Tumor Microenvironment
Source: Sci Rep. 2020 Jun 22;10:9955. doi: 10.1038/s41598-020-66471-2 (PMC7308356; doi:10.1038/s41598-020-66471-2)

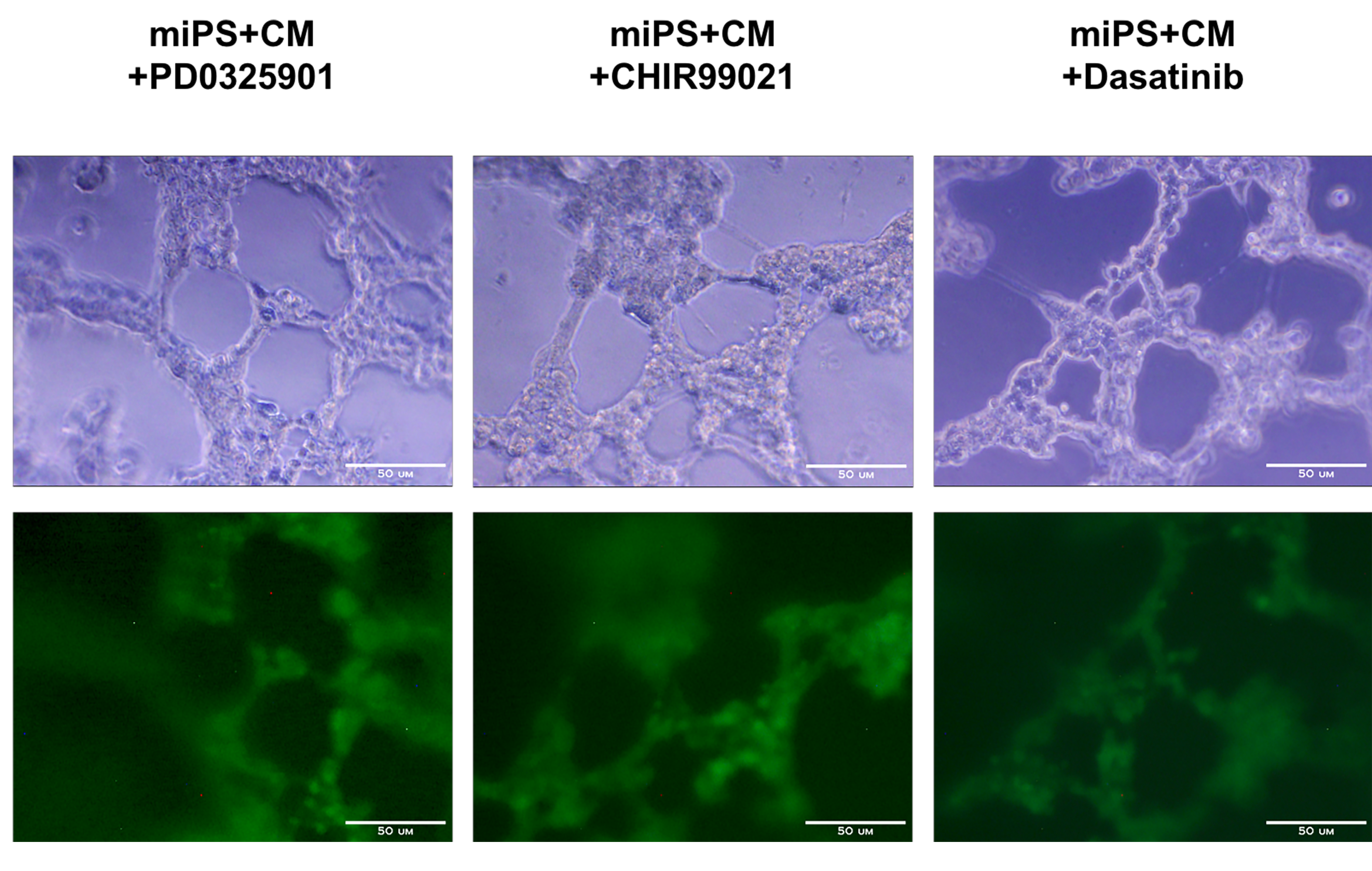

Supplement: Supplementary file 1 — Supplementary Figure S1. [file 41598_2020_66471_MOESM1_ESM.tif]

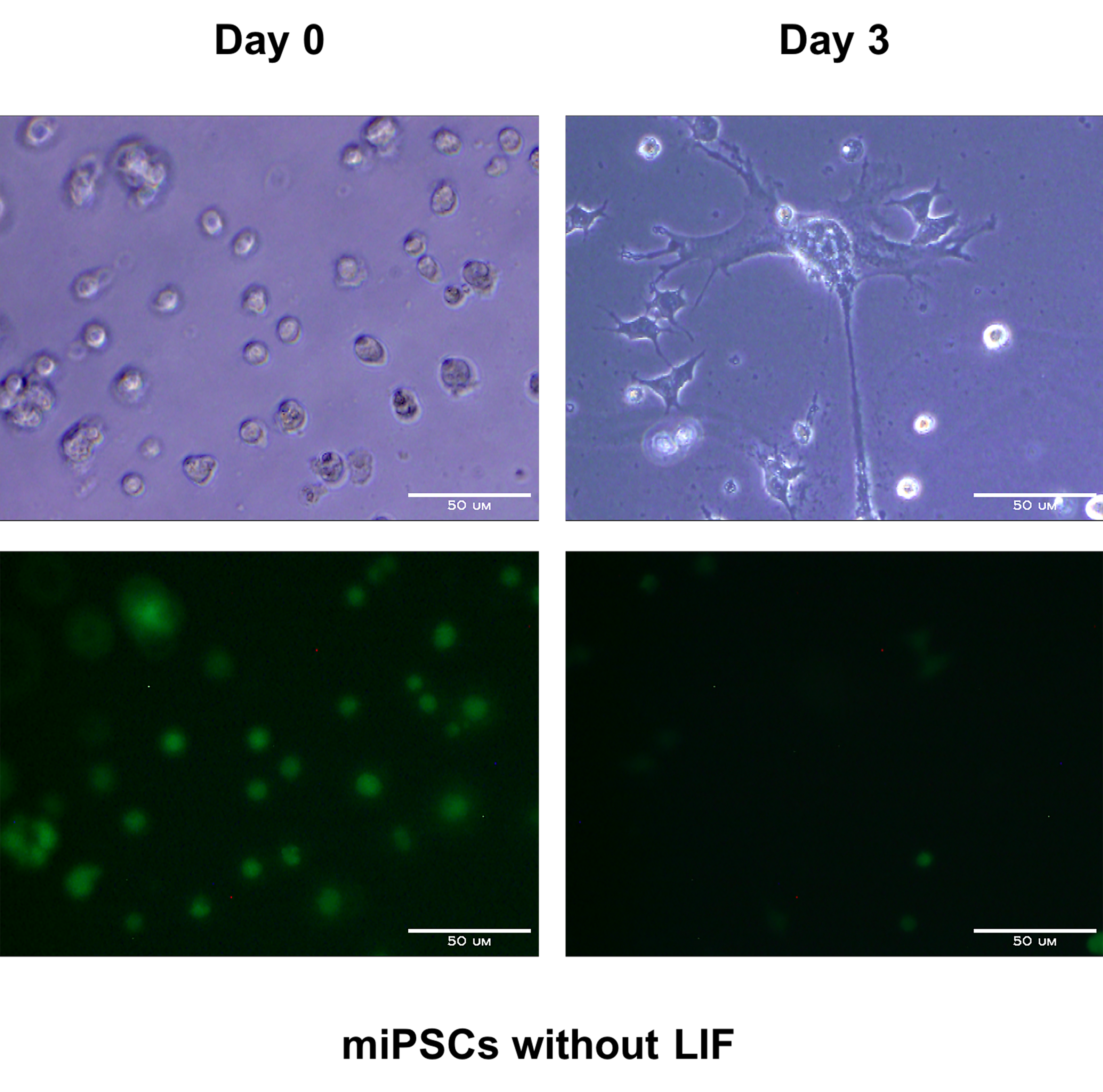

Supplement: Supplementary file 2 — Supplementary Figure S2. [file 41598_2020_66471_MOESM2_ESM.tif]

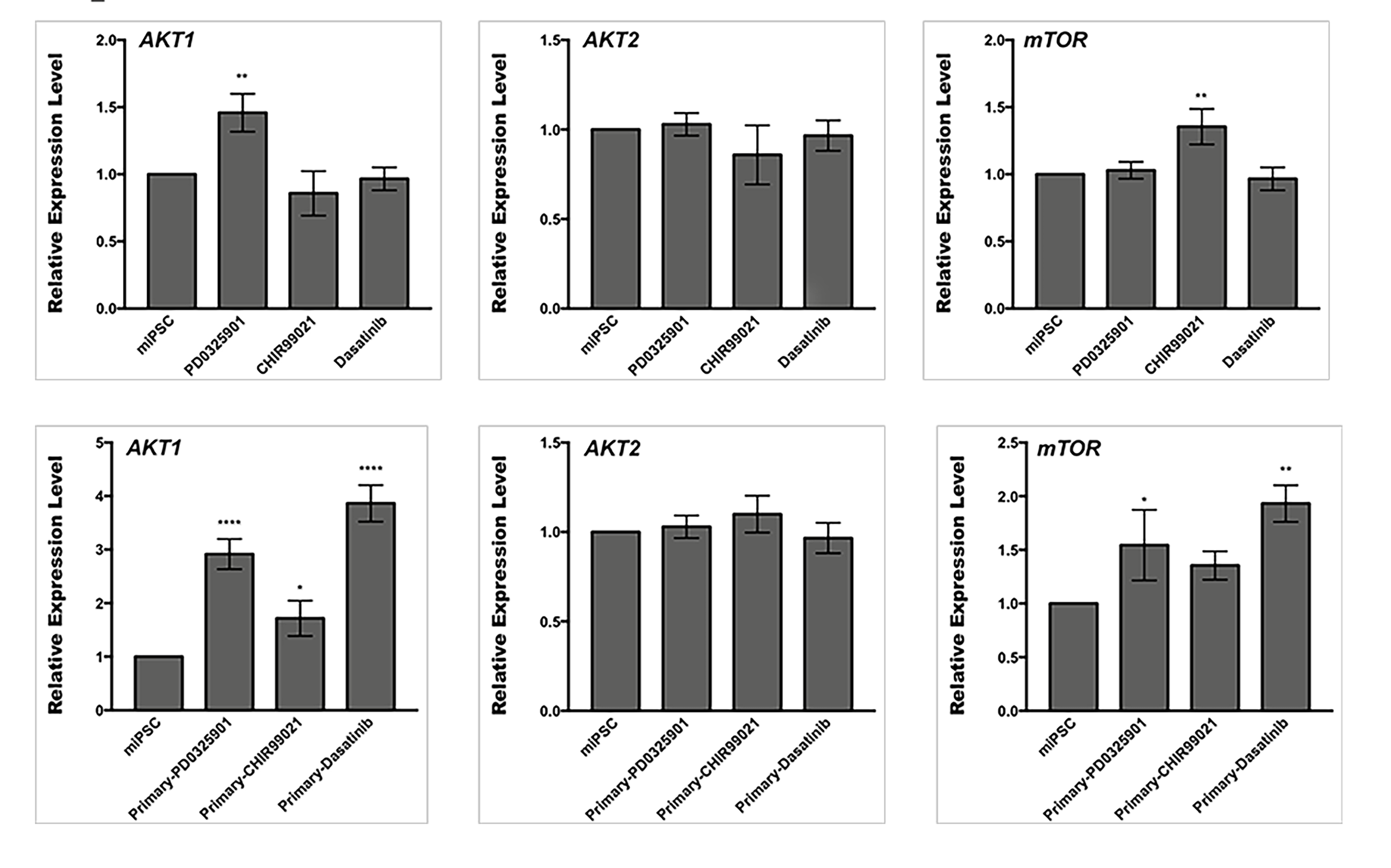

Supplement: Supplementary file 3 — Supplementary Figure S3. [file 41598_2020_66471_MOESM3_ESM.tif]
