# Supplementary material for: Signaling Inhibitors Accelerate the Conversion of mouse iPS Cells into Cancer Stem Cells in the Tumor Microenvironment
Source: Sci Rep. 2020 Jun 22;10:9955. doi: 10.1038/s41598-020-66471-2 (PMC7308356; doi:10.1038/s41598-020-66471-2)

Title

Signaling Inhibitors Accelerate the Conversion of mouse iPS Cells into Cancer Stem Cells in the Tumor Microenvironment

Authors

Juan Du[1], Yanning Xu[1,4], Saki Sasada[1], Aung Ko Ko Oo[1], Ghmkin Hassan[1], Hafizah Mahmud[1], Apriliana Cahya Khayrani[1], Md Jahangir Alam[1], Kazuki Kumon[1], Ryo Uesaki[1], Said M. Afify[1], Hager M. Mansour[1], Neha Nair[1], Maram H. Zahra[1], Akimasa Seno[1,2,3], Nobuhiro Okada[2], Ling Chen[4], Ting Yan[5], Masaharu Seno[1,2,3]

Affiliations

1 Department of Medical Bioengineering, Graduate School of Natural Science and Technology, Okayama University, Okayama 700-8530, Japan

2 Laboratory of Nano-Biotechnology, Graduate School of Interdisciplinary Science and Engineering in Health Systems, Okayama University, Okayama 700-8530, Japan

3 Okayama University Research Laboratory of Stem Cell Engineering in Detroit, IBio, Wayne State University, MI 48202, USA

4 Department of Pathology, Tianjin Central Hospital of Gynecology Obstetrics, Tianjin 300100, People's Republic of China

5 Department of Pathology, Shanxi Key Laboratory of Carcinogenesis and Translational Research on Esophageal Cancer, Shanxi Medical University, 030001 Taiyuan, PR China.

6 Division of Biochemistry, Chemistry Department, Faculty of Science, Menoufia University, Shebin El Kom-Menoufia 32511, Egypt.

Correspondence: Prof. Dr. Masaharu Seno, Department of Medical Bioengineering, Graduate School of Natural Science and Technology, Okayama University, Okayama 700-8530, Japan. Tel.: 81-86-251-8216, E-mail: mseno@okayama-u.ac.jp

Results of five standard experiments without inhibitors											
**Exp.**	**No. 1**		**No. 2**		**No. 3**		**No. 4**		**No. 5**		Overall
	GFPi[*1] *X10*^{3}	RFi[*2]	*GFPi X10*^{3}	RFi	*GFPi X10*^{3}	RFi	*GFPi X10*^{3}	RFi	*GFPi X10*^{3}	RFi	
	6.58	0.89	*8.43*	1.01	*6.59*	0.85	*9.03*	1.22	*9.08*	1.24	
	6.97	0.94	*13.88*	1.66	*10.16*	1.31	*7.03*	0.95	*10.3*	1.41	
	8.24	1.12	*6.39*	0.77	*8.05*	1.04	*5.63*	0.76	*6.16*	0.84	
	9.53	1.29	*5.71*	0.68	*5.76*	0.74	*7.08*	0.96	*7.38*	1.01	
	5.7	0.77	*7.41*	0.89	*10.12*	1.3	*8.61*	1.17	*5.79*	0.79	
	7.27	0.98	*8.33*	1	*6*	0.77	*6.92*	0.94	*5.21*	0.71	
**average**[*3]	*7.38*	1	*8.36*	1	*7.78*	1	*7.38*	1	*7.32*	1	1
**error**	*1.34*	0.18	*2.91*	0.35	*1.99*	0.26	*1.24*	0.17	*2*	0.27	0.24[*4]

GFP fluorescence intensity (GFPi[*1]) depicted the read from each well of miPSCs present in CM and the average of the reads were calculated for each experiment. Then the relative fluorescence intensity (RFi[*2]) was depicted in the table after division by each average value[*3]. From the table above the thresholds to distinguish the results were determined as 1.00±0.24[*4] to show the following: positive > 1.24; 0.76 ≤ no significant difference ≤1.24; negative < 0.76.

Table S 1. Thresholds to distinguish positive and negative results.

Name	CAS No.	Company	Target of signaling pathway	GFP fluorescence (P, N, NSD)	optimal concentration (μM)
Masitinib	790299-79-5	AdooQ BioScience	Kit, PDGFα/β	P	6.25
Quizartinib	950769-58-1	AdooQ BioScience	FLT3	P	1.25
Alk5 Inhibitor II	446859-33-2	STEMGENT	TGF-β	P	10
Bafetinib (INNO-406)	859212-16-1	Selleck Chemicals	BCR/ABL	P	1.25
BIRB-796	285983-48-4	AdooQ BioScience	p38-MAPK	P	5
CHIR99021	252917-06-9	Selleck Chemicals	GSK3	P	2.5
Cycbpamine	4449-51-8	STEMGENT	Hedgehog	P	5
Dasatinib	302962-49-8	AdooQ BioScience	BCR/ABL, Src	P	1.25
DCC-2036 (Rebastinib)	1020172-07-9	Selleck Chemicals	Src, ABL1	P	2.5
DMAXX (vadimezan)	117570-53-3	Selleck Chemicals	VDAs	P	0.63
Fingolimod (FTY720)	162359-56-0	Selleck Chemicals	TGF–β	P	1
GZD824	1421783-64-3	Selleck Chemicals	BCR/ABL	P	0.63
imatinb mesylate	220127-57-1	Selleck Chemicals	BCR/ABL	P	25
Imatinib	152459-95-5	AdooQ BioScience	BCR/ABL	P	6.25
KAAD-Cycbpamine	306387-90-6	STEMGENT	GLI1	P	5
PD0325901	391210-10-9	Selleck Chemicals	MEK	P	5
ponatinib	943319-70-8	Selleck Chemicals	ABL, PDGFR, VEGFR, FGFR, Src	P	0.63

R(+)Bay K8644	98791-67-4	Selleck Chemicals	L-type Ca2+ channel activator1,2	P	5
R406	841290-80-0	Cayman Chemical Company	Syk	P	10
Thiazovivin	1226056-71-8	STEMGENT	RHO/ROCK	P	15
2-Methoxyestradiol (2-MeOE2)	362-07-2	Selleck Chemicals	TGF-β	NSD	—
AG-013736	319460-85-0	Selleck Chemicals	VEGFR	NSD	—
AST-487	630124-46-8	Adipogen Life Sciences	FLT3	NSD	—
AT9283	896466-04-9	Selleck Chemicals	Aurora B	NSD	—
AZD-6244/ARRY-886	606143-52-6	Selleck Chemicals	VEGFR	NSD	—
BI-2536	755038-02-9	AdooQ BioScience	Plk1	NSD	—
BIBF-1120 (derivative)	656247-17-5	AdooQ BioScience	VEGFR, PDGF, FGFR	NSD	—
BIBW-2992	850140-72-6	AdooQ BioScience	Her2, EGFR	NSD	—
BIO	667463-62-9	Cayman Chemical Company	GSK3	NSD	—
BIX01294	935693-62-2	STEMGENT	Histone lysine methyltransferase	NSD	—
BMS-345541	547757-23-3	AdooQ BioScience	NF-κB	NSD	—
BMS-387032/SNS-032	345627-80-7	AdooQ BioScience	CDKs	NSD	—
BMS-540215	649735-46-6	AdooQ BioScience	VEGFR	NSD	—
CEP-701	111358-88-4	Focus Biomolecules	FLT3, JAK2, TrkA	NSD	—
CHIR-258/TKI-258	405169-16-6	AdooQ BioScience	FGFR	NSD	—
CHIR-265/RAF-265	927880-90-8	AdooQ BioScience	RAF, VEGFR	NSD	—
CI-1033	289499-45-2	Cayman Chemical Company	ErbBR	NSD	—

Crizotinib	877399-52-5	LC Laboratories, Inc	ALK	NSD	—
Danusertib (PHA-739358)	827318-97-8	Selleck Chemicals	FGFR, ABL	NSD	—
Degrasyn (WP1130)	856243-80-6	Selleck Chemicals	BCR/ABL	NSD	—
DMF	1968/12/2	SIGMA	prepare solution	NSD	—
DMSO	67-68-5	SIGMA	prepare solution	NSD	—
Dorsomorphin	866405-64-3	STEMGENT	AMPK	NSD	—
Doxycycline hyclate	24390-14-5	Selleck Chemicals	Inhibit the inflammatory	NSD	—
Erlotinib	183321-74-6	Cayman Chemical Company	EGFR	NSD	—
FG-4592	808118-40-3	Selleck Chemicals	HIF-PH	NSD	—
Forskolin	66428-89-5	STEMGENT	MAPK	NSD	—
GDC-0879	905281-76-7	AdooQ BioScience	CDK	NSD	—
GDC-0941	957054-30-7	AdooQ BioScience	CLASS I PI3K	NSD	—
Gefitinib	184475-35-2	AdooQ BioScience	EGFR	NSD	—
GSK-690693	937174-76-0	Synkinase Pty Ltd	AK1/2/3	NSD	—
GW-2580	870483-87-7	Cayman Chemical Company	cFMSR	NSD	—
HKI-272	698387-09-6	AdooQ BioScience	EGFR	NSD	—
IDE-1	11160927-48-9	STEMGENT	Inducer ofdefinitive endoderm	NSD	—
IDE-2	N/A	STEMGENT	TGF-β	NSD	—
JNJ-28312141	885692-52-4	AdooQ BioScience	CSF1R	NSD	—
KW-2449	1000669-72-6	AdooQ BioScience	FLT3, STAT5	NSD	—

	Lapatinib	231277-92-2	AdooQ BioScience	Her2	NSD	—
	LY-317615	170364-57-5	AdooQ BioScience	PKC-β	NSD	—
	LY-333531	169939-93-9	Cayman Chemical Company	PKC-β	NSD	—
	MLN-120B	783348-36-7	MedChemexpress Co., limite	IKK-β	NSD	—
	MLN-518	387867-13-2	AdooQ BioScience	FLT3, cKit, PDGFR	NSD	—
	MLN-8054	869363-13-3	AdooQ BioScience	Aurora A	NSD	—
	Nilotinib	641571-10-0	AdooQ BioScience	BCR/ABL	NSD	—
	PD-173955	260415-63-2	Synkinase Pty Ltd	Src/ Abl	NSD	—
	PHA-665752	477575-56-7	AdooQ BioScience	c-Met/HGF/SF	NSD	—
	PI-103	371935-79-4	AdooQ BioScience	PI3K	NSD	—
	Pifithrin-alpha	63208-82-2	STEMGENT	p53	NSD	—
	plinabulin(NPI-2358)	714272-27-2	Selleck Chemicals	Angiogenesis and Tumor vasculature	NSD	—
	PLX-4720	918505-84-7	Cayman Chemical Company	B-raf	NSD	—
	PP-242	1092351-67-1	AdooQ BioScience	mTOR	NSD	—
	PTK-787	212141-51-0	AdooQ BioScience	VEGFR	NSD	—
	pumorphamine	483367-10-8	STEMGENT	Hedgehog	NSD	—
	R547	741713-40-6	AdooQ BioScience	CDKs	NSD	—
	RG108	48208-26-0	STEMGENT	DNA methyltransferase	NSD	—
	ROCK II Inhibitor	N/A	STEMGENT	ROCK	NSD	—
34	Saracatinib (AZD0530)	379231-04-6	Selleck Chemicals	Src	NSD	—

SB431542	301836-41-9	STEMGENT	TGF-β	NSD	—
SC1(Pluripotin)	839707-37-8	STEMGENT	ERK1	NSD	—
SGX-523	1022150-57-7	AdooQ BioScience	MET	NSD	—
Sorafenib	475207-59-1	AdooQ BioScience	VEGFR	NSD	—
Staurosporine	62996-74-1	Cayman Chemical Company	Prevent ATP binding to the kinase	NSD	—
SU-14813	627908-92-3	AdooQ BioScience	VEGFR, PDGF, FGFR	NSD	—
Sunitinib	341031-54-7	Cayman Chemical Company	VEGFR	NSD	—
TAE-684	761439-42-3	Synkinase Pty Ltd	ALK	NSD	—
TG-100-115	677297-51-7	AdooQ BioScience	PI3K	NSD	—
TG-101348	936091-26-8	AdooQ BioScience	JAK	NSD	—
Tranylcypromine hydrochloride	1986-47-6	STEMGENT	MAO	NSD	—
Vandetanib	443913-73-3	Cayman Chemical Company	VEGFR,EGFR	NSD	—
Veliparib (ABT-888)	912444-00-9	Selleck Chemicals	PARP	NSD	—
Wnt inhiitor iwp-2	686770-61-6	STEMGENT	Wnt	NSD	—
Wnt inhiitor iwp-3	N/A	STEMGENT	Wnt	NSD	—
Wnt inhiitor iwp-4	N/A	STEMGENT	Wnt	NSD	—
Y27632	146986-50-7	STEMGENT	ROCK	NSD	—
A-674563	552325-73-2	AdooQ BioScience	PKA, CDK2,AKT	N	12.5
A83-01	909910-43-6	Selleck Chemicals	TGF-β, ALK5	N	10
ABT-869	796967-16-3(4)	AdooQ BioScience	RTK, VEGF,PDGF	N	6.25

All-Trans Retinoic Acid	302-79-4	Selleck Chemicals	Ligand for the retinoic acid receptor	N	1
AMG-706	857876-30-3	AdooQ BioScience	VEGFR	N	10
DAPT	208255-80-5	STEMGENT	Notch	N	10
IOX2	931398-72-0	Selleck Chemicals	PHD	N	0.63
IPA3	42521-82-4	STEMGENT	Pak1	N	2.5
LDN-193189	1062368-24-4	STEMGENT	BMP	N	5
Nibtinib (AMN-107)	641571-10-0	Selleck Chemicals	ERK1/2, PDGF	N	0.31
NVP-BHG712	940310-85-0	Selleck Chemicals	RTK	N	15
Olaparib (AZD2281, Ku-0059436)	763113-22-0	Selleck Chemicals	BRCA1, BRCA2	N	1
PD173034	219580-11-7	STEMGENT	FGFR	N	5
Pifithrin-mu(u)	64984-31-2	STEMGENT	p53	N	10
SMO antagonist	N/A	STEMGENT	Hedgehog	N	5
Tanespimycin (17-AAG)	75747-14-7	Selleck Chemicals	Her2, AKT	N	0.5

P: Positive compound, N: Negative ompound, NSD: No Significant Difference compound

Table S 2. The list of chemical compounds (110).

NO.	Names	ACCESSION	Forward Primer Sequence 5’- 3’	Reverse Primer Sequence 5’- 3’
1	mouse Oct3/4 endogeneous	NM_013633.2	*TCTTTCCACCAGGCCCCCGGCTC*	*TGCGGGCGGACATGGGGAGATCC*
2	mouse Sox2 endogeneous	NM_011443.3	*TAGAGCTAGACTCCGGGCGATGA*	*TTGCCTTAAACAAGACCACGAAA*
3	mouse Klf4 endogeneous	NM_010637.3	*GGACTTACAAAATGCCAAGGGGTG*	*TCGCTTCCTCTTCCTCCGACACA*
4	mouse c-myc endogeneous	NM_010849.4	*TGACCTAACTCGAGGAGGAGCTGGAATC*	*AAGTTTGAGGCAGTTAAAATTATGGCTGAAGC*
5	mouse Oct3/4 total	NM_013633.2	*CTGAGGGCCAGGCAGGAGCACGAG*	*CTGTAGGGAGGGCTTCGGGCACTT*
6	mouse Sox2 total	NM_011443.3	*GGTTACCTCTTCCTCCCACTCCAG*	*TCACATTGTCGACAGGGGCAG*
7	mouse Klf4 total	NM_010637.3	*CACCATGGACCCGGGCGTGGCTGCCAGAAA*	*TTAGGCTGTTCTGGGCCGGGGCCACGA*
8	mouse c-myc total	NM_010849.4	*CAGAGGAGGAACGAGCTGAAGCGC*	*TTATGCACCAGAGTTTCGAAGCTGTTCG*
9	mouse Oct3/4 transgene	M34381.1	*TTGGGCTAGAGAAGGATGTGGTTC*	*TTATCGTCGACCACTGTGCTGCTG*
10	mouse Sox2 transgene	NM_003106.4	*GGTTACCTCTTCCTCCCACTCCAG*	*TTATCGTCGACCACTGTGCTGCTG*
11	mouse Klf4 transgene	XM_021200389.1	*GCGAACTCACACAGGCGAGAAACC*	*TTATCGTCGACCACTGTGCTGCTG*
12	mouse c-myc transgene	NM_010637.3	*CAGAGGAGGAACGAGCTGAAGCGC*	*TTATCGTCGACCACTGTGCTGCTG*
13	mouse Nanog	NM_028016.3	*AGGGTCTGCTACTGAGATGCTCTG*	*CAACCACTGGTTTTTCTGCCACCG*
14	Pik3ca	NM_008839.2	*GCCACAGACACTACTGCGTA*	*CACCGAACAGCAAAACTCCG*
15	Pik3cb	NM_029094.3	*CTGATTTTACGGCGGCATGG*	*TGAGGGCCTCGTCAAACTTC*
16	Pik3cg	NM_001146201	*ACCTGTGCCTTCTGCCTTAC*	*TGCGGCCTGAAACTTTTCTTC*
17	Pik3r1	NM_001024955.2	*AGCGGAGAACCTATTGCGAG*	*ACTTCGCCGTCTACCACTAC*
18	Pik3r5	NM_177320.2	*AAGTCCTTTGTCAGCAGTCCC*	*CTGGTAAACCTGCAGCAACAC*
19	Pik3r6	NM_001004435.3	*TGAGACGACCACATCCTCCC*	*TCCACATGCCCTGATTGCTC*
20	Pik3ap1	NM_031376.4	*GAAGGCCATTTCTGAAGATTCTGG*	*TCTCGTCCAGCTTGCATCTC*
21	Pten	NM_001304718.2	*TGAAGACCATAACCCACCACAG*	*AGCATCTTGTTCTGTTTGTGGAAG*

Table S 3. List of Primers Used in the Experiments.

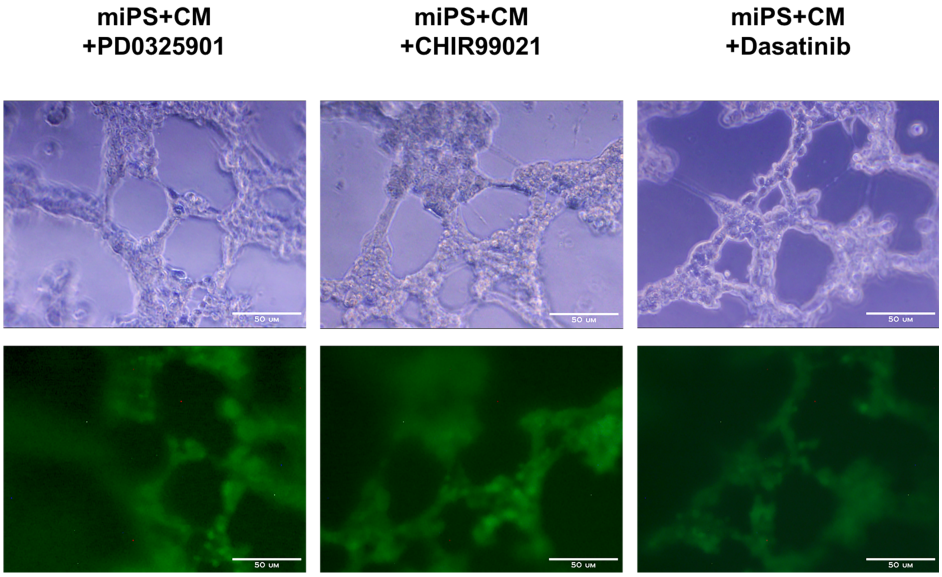

.

Fig. S1 Tube formation assays in miPSCs and other three converted cells after 20h of culture on Matrigel. Differentiation potential is another property of CSCs, along with self-renewal. miPSCs and other three converted cells were assessed for the potential to differentiate into endothelial-like cells forming capillary-like tubes on Matrigel. Capillary-like tubes were formed by these cells indicating pro-angiogenic properties in tumorigenesis. These cells showed high potential of tube formation.

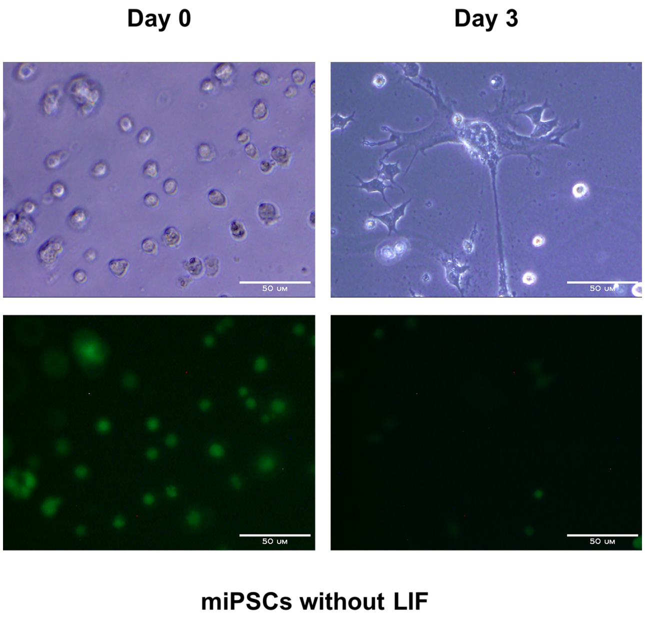

Fig. S2 miPSCs in adherent culture without LIF. Left: miPSCs with high expression of GFP. Right: after 3 days, most of the cells differentiated and no expression of GFP. After 1 week no cells survived.

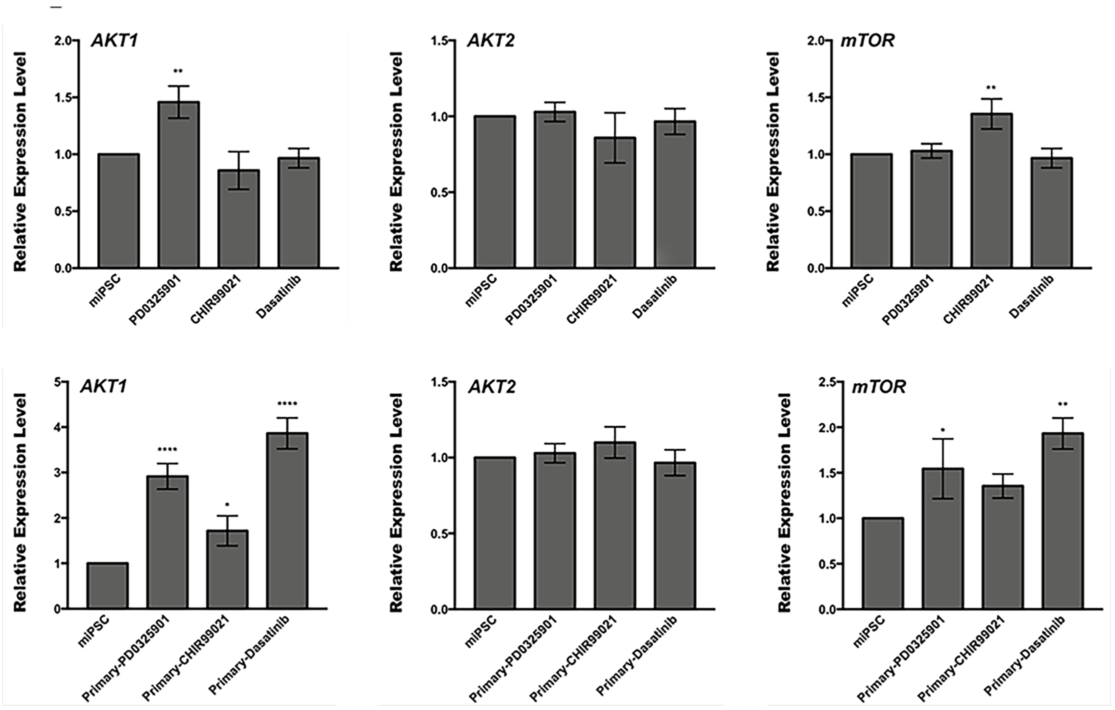

Fig. S3 RT-qPCR analysis of AKT1, AKT2 and mTOR.

Figure 6b (Full unedited gels of Western blots)

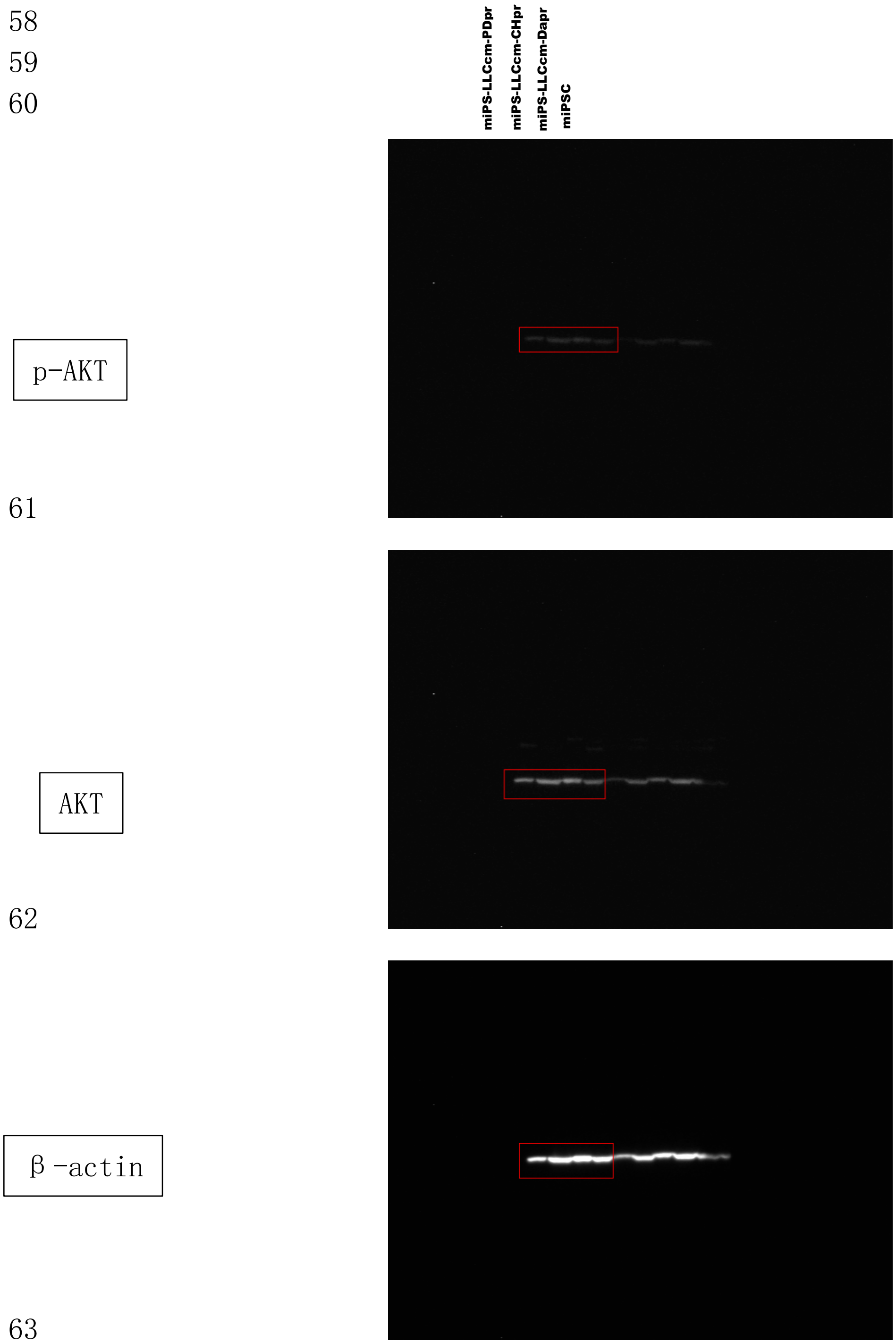

Figure 6c (Full unedited gels of Western blots)

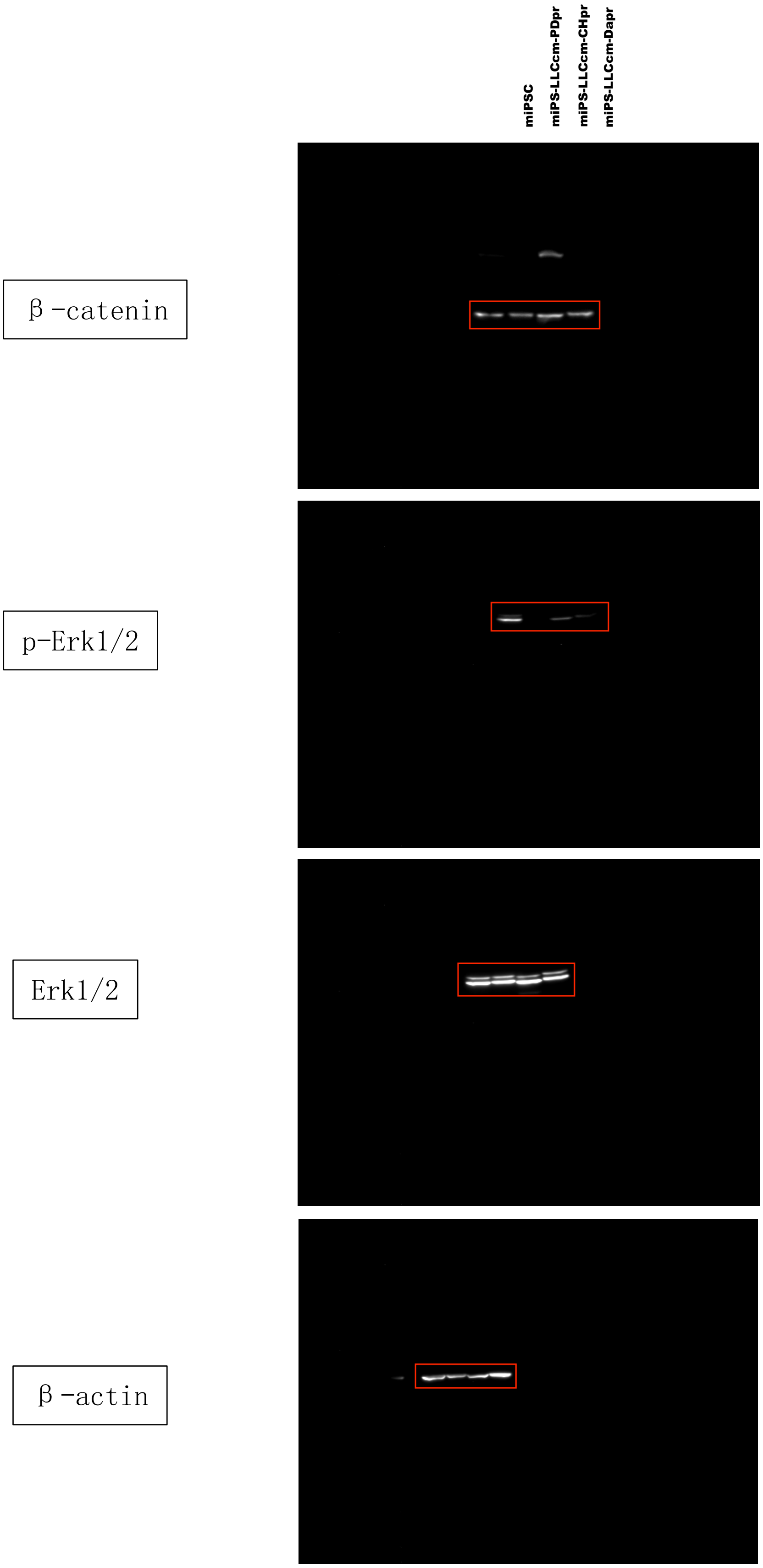

Supplement: Supplementary file 4 — Supplementary Information. [file 41598_2020_66471_MOESM4_ESM.pdf]
